# Resistance of Lepidopteran Pests to *Bacillus thuringiensis* Toxins: Evidence of Field and Laboratory Evolved Resistance and Cross-Resistance, Mode of Resistance Inheritance, Fitness Costs, Mechanisms Involved and Management Options

**DOI:** 10.3390/toxins16070315

**Published:** 2024-07-12

**Authors:** Muhammad Babar Shahzad Afzal, Mamuna Ijaz, Naeem Abbas, Sarfraz Ali Shad, José Eduardo Serrão

**Affiliations:** 1 Beekeeping & Hill Fruit Pests Research Station, Rawalpindi 46000, Pakistan; shahzad.babar35@gmail.com; 2 Department of Entomology, Faculty of Agricultural Sciences and Technology, Bahauddin Zakariya University, Multan 60800, Pakistan; mamunaejaz65@gmail.com; 3 Pesticides and Environmental Toxicology Laboratory, Department of Plant Protection, College of Food and Agriculture Sciences, King Saud University, P.O. Box 2460, Riyadh 11451, Saudi Arabia; 4 Department of General Biology, Federal University of Vicosa, Vicosa 36570-900, MG, Brazil; jeserrao@ufv.br

**Keywords:** resistance evolution, fitness costs, mechanisms, resistance management, transgenic crops, insecticidal proteins

## Abstract

*Bacillus thuringiensis* (*Bt*) toxins are potential alternatives to synthetic insecticides for the control of lepidopteran pests. However, the evolution of resistance in some insect pest populations is a threat and can reduce the effectiveness of *Bt* toxins. In this review, we summarize the results of 161 studies from 20 countries reporting field and laboratory-evolved resistance, cross-resistance, and inheritance, mechanisms, and fitness costs of resistance to different *Bt* toxins. The studies refer mainly to insects from the United States of America (70), followed by China (31), Brazil (19), India (12), Malaysia (9), Spain (3), and Australia (3). The majority of the studies revealed that most of the pest populations showed susceptibility and a lack of cross-resistance to *Bt* toxins. Factors that delay resistance include recessive inheritance of resistance, the low initial frequency of resistant alleles, increased fitness costs, abundant refuges of non-*Bt,* and pyramided *Bt crops*. The results of field and laboratory resistance, cross-resistance, and inheritance, mechanisms, and fitness cost of resistance are advantageous for predicting the threat of future resistance and making effective strategies to sustain the effectiveness of *Bt* crops.

## 1. Introduction

*Bacillus thuringiensis* (*Bt*)-transformed plants are globally used as cultivated crops [1,2]. *Bt* toxins are environmentally benign and more selective on target pests; moreover, crop dependency upon the application of conventional insecticides is also reduced, thus they have revolutionized the agriculture sector [3,4]. The *Bt* toxins ingested by insects are proteolytically activated in the insect gut lumen and the activate toxins interact with membrane proteins in lepidopteran midgut cells, causing the formation of ionic pores in the cell membrane and inducing cell death [5]. As *Bt* crops help to reduce the incidence of attack by lepidopteran pests, the yield potential of cotton and maize increases many fold and higher profits may incur [6,7,8]. The *Bt* crops have been cultivated on >560 million hectares area worldwide since 1996 [2,9]. *Bt* cotton has been planted on 69 million hectares of area globally, with 79–95% area in Australia, China, India, Pakistan, and the United States [10], while 67% of the total area of *Bt* maize is planted in the United States [1].

Some lepidopteran insect pests present tolerance to *Bt* toxin effects and develop resistance, which may be a risk to the sustained success of *Bt* cotton and maize. The *Bt* resistance in lepidopteran pests of cotton and maize has been reported globally and is summarized in this review. We described the observed pattern of field-evolved and laboratory-selected resistance, the mechanism of resistance, and the fitness costs of resistance reported against *Bt* toxins during the period of 2000–2018. Due to time constraints, we cannot extend this review further at present. However, we plan to write another review paper in the future, focusing on the resistance scenario from 2019 to 2024 in various pests against *Bt* toxins. In this review, 24 cases of field-evolved resistance to 12 *Bt* toxins and 37 instances of cross-resistance to 28 *Bt* toxins in nine lepidopteran species were reported. Additionally, the review described 63 cases of laboratory-selected resistance and 58 cases of inheritance of resistance to 15 toxins in 11 species, 57 cases of the mechanism of resistance to 13 toxins in 11 species, and 36 reports of the fitness cost of resistance to 11 toxins in 12 species. These results may provide insights to develop and improve proactive strategies for the resistance management of various pest species to *Bt* toxins.

## 2. *Bt* Resistance Management

After the first report of *Bt* resistance by McGaughey [11] researchers worldwide have attempted to elucidate the cross-resistance, genetics, fitness costs and mechanisms triggering the *Bt* resistance in some Lepidoptera pests. These efforts are vital if novel strategies are to be developed to use currently available *Bt* toxins effectively and efficiently for the prevention of resistance development.

Many strategies for *Bt* resistance management have been proposed, including high toxin dose in combination with refugia, pyramiding/stacking (use of multiple toxins simultaneously), and crop rotations. High dose-refuges that contain toxin-free host plants near the *Bt* crops might play an important role in insect resistance management (IRM) plans. Using a 20% refuge of non-transgenic crops treated with non-*Bt* foliar insecticides or a 4% non-transgenic crop, delays the development of resistance in cotton and maize pests [12,13]. These refuges allow the influx of susceptible individuals that dilute the occurrence of resistance genes. The success of this approach occurs in the recessive mode of inheritance in combination with pest biology [14]. Ultra-high *Bt* toxin doses in combination with the refugia may also kill the heterozygote insects carrying the resistant alleles. So, the individuals would be more likely to mate with non-resistant insects, with a possible decrease in homozygote individuals [14]. Data about cross-resistance, mode of inheritance, fitness costs, and mechanisms of resistance involved provide assistance for the detection, monitoring, modeling, and future risk assessment of resistance [15].

Pyramiding *Bt* toxins is a recent technology used to delay the inception of resistance for plants having transgenic genes of two different toxins, when cross resistance is absent [2,16]. Other promising factors that favor the pyramiding crop are low initial resistance alleles frequency and fitness costs, associated with a recessive inheritance [16,17]. Cross-resistance between Cry 1Ac and Cry 2Ab is unlikely to occur due to the different amino acids and binding sites in the insect midgut [18,19]. So, pyramiding the most feasible *Bt* toxins that are expected to have no cross-resistance is a good approach to IRM practices. This technology provides a broader spectrum of activity against lepidopterans, enhances control of caterpillars that are susceptible to single *Bt* toxin transgenic plants, and provides better opportunities for managing insect resistance to Cry proteins [20].

Reducing the selection pressure by restricting the expression of *Bt* proteins to susceptible plant tissue or at a certain threshold level may also be an important management strategy. Expression of *Bt* toxins could be induced by wound inducible promoters after insect feeding or by the application of chemicals [21]. For example, it has been proposed that cotton plants undergo low yield loss when *Bt* toxin expression is restricted to only young bolls, which stimulates *Heliothis* larvae to feed on the other plant parts [22].

Resistance development can be mitigated by using comprehensive and systematic resistance management strategies. With pyramiding and high dose-refuge, especially in the case of a recessive genetic basis of resistance, non-insecticidal control (pheromone mating disruption, crop rotation, and suppression of overwintering stages of insects though soil or stalk disruption) may be added in the transgenic and non-transgenic cropping systems to manage and monitor pest population growth. Thus, crops that express *Bt* toxins are ensured to be a valuable tool in IRM programs.

## 3. Field-Selected Resistance to *Bt* Toxins

Field-selected resistance is a genetic-based decrease in susceptibility of a pest population due to its exposure to the toxin in the field [23]. Insecticide resistance has been suggested to be a pre-adaptive phenomenon in the sense that, before an organism is exposed to the toxin, there are few individuals in the population with one or more resistance genes that allow them to survive the toxin exposure [24,25]. Then, field resistance occurs after continuous exposure of the insect population to a toxin, which may increase the number of resistant alleles in the next generations. Insect pest control concerns due to field-selected resistance vary from none to severe according to the number of resistant individuals, the extent of the resistance increase, the density and geographical distribution of resistant populations, and the accessibility of alternative control methods [26].

Monitoring of *Bt* field-selected resistant populations may be conducted in laboratory tests to define baseline data and to ensure effective proactive management of insecticide resistance in target pests [26]. For this purpose, the field populations of different lepidopteran pests of cotton and corn are collected and exposed to different *Bt* crystals (Cry) and vegetative insecticidal proteins (Vip). These field populations are sampled from *Bt* and non-*Bt* crops to detect the field-selected resistance. The resistance was classified into categories, and the resistance ratios (RRs) were estimated as [27]:

RR = LC_50_ of field strain/LC_50_ of reference susceptible strain

where LC_50_ is the lethal concentration of toxin, killing 50% of insects tested under a controlled environment.

Based on the RR, we defined field resistance into three categories: (i) no to very low resistance, if the RR of the tested population is 1–10, (ii) low to moderate resistance, if the RR is 11–50, and (iii) high to very high resistance if the RR > 51. In the very low resistance, the amount of resistant insects in the population is not sufficient to reduce the efficacy of the *Bt* toxins for pest control. On the other hand, in the low–moderate resistance, there is a decrease in the efficacy of the *Bt* toxins to control field populations, indicating the necessity of resistance management. Finally, if the field population reaches very high resistance, the efficacy of the *Bt* toxins in the field fails and the resistance management strategy must be improved.

In this study, we reviewed 24 cases of resistance monitoring in nine species of main lepidopteran pests that were targeted by 12 *Bt* toxins in nine countries during the years 2000–2018 (Table 1).

We found reports in China of very high resistance to Cry1C for *Plutella xylostella* [28], no–very low resistance to Cry1Ac for *Pectinophora gossypiella* [29,30,31] and very low–moderate resistance to Vip3Aa11 for *Helicoverpa armigera* [32]. In India, *P. gossypiella* presented no–moderate resistance to Cry1Ac and Cry1Ac+Cry2Ab2 [33] and *H. armigera* showed no–very high resistance to Cry1Ac [34,35] and no–very low resistance to Cry2Ab [36], whereas in Pakistan, *H. armigera* showed very high resistance to Cry1Ac [37]. In West Africa, this pest presented very low–moderate resistance to Cry1Ac and no–very low resistance to Cry2Ab2 toxins [38]. In the USA, moderate resistance to Cry2Ab, very high resistance to Cry1Ac, no–high resistance to Vip3A, very low resistance to Cry1Fa, and moderate resistance to MVP II were reported for *H. zea* [39,40,41,42]. Very low resistance to Cry1Ac and very low–very high resistance to Vip3A were reported for *H. virescens* [40,41]. The *Spodoptera frugiperda* field populations have been reported to develop high–very high resistance to Cry1F [43,44,45] and low resistance to Cry2Ab2 [46] in the USA. In contrast, no–very low resistance to Cry1F was found in different *S. frugiperda* populations from the USA [47]. For *Diatraea saccharalis*, very low resistance to Cry1F, moderate–very high resistance to Cry1Ac, and high–very high resistance to Cry1Ab and Cry1Aa have been reported [48]. In Brazil, no–very low resistance to Vip3Aa20 and Cry1Ac for *H.zea* and *H. armigera* [49,50,51], very low–very high resistance to Cry1F for *S. frugiperda* [52,53,54], and no–very low resistance to Vip3Aa20 for *S. frugiperda* and *D. saccharalis* have been reported. *Ostrinia furnacalis* and *O. nubilalis* have shown no–low resistance to Cry1Ab maize in the Philippines, Germany, and Europe [55,56,57].

Those different levels of resistance to the same *Bt* toxin by different populations of the same species, to different toxins, and among different pests reinforce the necessity of laboratory monitoring of field populations to the bio-insecticide to predict possible selection of field resistance and the use of pesticide management to avoid very high resistance.

**Table 1:** Field-evolved resistance to *Bacillus thuringiensis* toxins in different lepidopteran pests from 2000–2018.

Species	Country	Crop	Toxin	RR	Resistance Level	Reference
*Plutella xylostella*	China	Cabbage	Cry1C	740	Very high	[28]
*Pectinophora gossypiella*	India	Cotton	Cry1Ac	1–47	No–moderate	[33]
			Cry1Ac+Cry2Ab2	1–2	No resistance	
	China	Cotton	Cry1Ac	1.4–14.6	No–low	[58]
	USA	Tobacco	Cry2Ab	>27	Moderate	[39]
		Maize	Cry1Ac	578	Very high	[41]
		Cotton	Vip3A	1–75	No–high	[40]
		Maize	Cry1Fa	8.4	Very low	[42]
		Maize	MVP II	34.5	Moderate	
	Brazil	Maize	Vip3Aa20	1.25–5.25	No–very low	[49]
*Heliothis virescens*	USA	Maize	Cry1Ac	3.2	Very low	[41]
	USA	Cotton	Vip3A	2–133	Very low–very High	[40]
	Brazil	Cotton	Cry1Ac	0.27–0.62	No resistance	[51]
*Helicoverpa armigera*	China	Cotton	Cry1Ac	0.42–5	No–very low	[29]
	India	Cotton	Cry1Ac	1.4–119	No–very high	[34]
	India	Cotton	Cry1Ac	1.1–96.7	No–high	[35]
	India	Cotton	Cry2Ab	1.1–14	No–very low	[36]
	West Africa	Cotton	Cry1Ac	4.7–44	Very low–moderate	[38]
			Cry2Ab2	1.2–9.9	No–very low	
	China	Cotton	Cry1Ac	11	Low	[30]
	Pakistan	Cotton	Cry1Ac	580	Very high	[37]
	China	Cotton	Cry1Ac	11	Low	[30]
	Brazil	Soybean	Cry1Ac	0.1–1.5	No resistance	[50]
	Brazil	Maize	Vip3Aa20	1.2–2.8	No–very low	[49]
	China	Cotton	Vip3Aa11	3.3–24.7	Very low–moderate	[32]
	China	Cotton	Cry1Ac	0.8–3.6	No–very low	[31]
*Spodoptera frugiperda*	USA	Maize	Cry1F	>1000	Very high	[43]
	USA	Maize	Cry2Ab2	>15	Low	[46]
	Brazil	Maize	Cry1F	>5525	Very high	[52]
	Brazil	Maize	Vip3Aa20	1.02–6.62	No–very low	[59]
	USA	Maize	Cry1F	>85.4	High	[44]
	USA	Maize	Cry1F	7717	Very high	[45]
	Brazil	Maize	Cry1Fa	>10	Low	[53]
	USA	Maize	Cry1F	1.29–7.92	No–very low	[47]
	Brazil	Maize	Cry1Ab	Absent	Absent	[60]
	Brazil	Maize	Cry1F	4.1->85	Very low–high	[44,54]
*Diatraea saccharalis*	USA	Maize	Cry1Ab	53–526	High–very high	[48]
			Cry1Aa	71–292	High–very high	
			Cry1Ac	30–248	Moderate–very High	
			Cry1F	1.5–5.2	Very low	
	Brazil	Maize	Vip3Aa20	1.22–6.01	No–very low	[59]
*Ostrinia furnacalis*	Philippines	Maize	Cry1Ab	1.5–5.6	Very low	[55]
*Ostrinia nubilalis*	Germany	Maize	Cry1Ab	8.5	Very low	[56]
	Europe	Maize	Cry1Ab	1–14	No–Low	[57]

RR (resistance ratio) = LC_50_ of field strain ÷ LC_50_ of susceptible strain.

## 4. Laboratory-Selected Resistance to *Bt* Toxins

Genetic-based increases in resistance to an insecticide in a population due to its continuous exposure in the laboratory is termed laboratory-selected resistance [23]. The laboratory-selected resistant strains are useful for evaluating pesticide risk assessment, cross-resistance, stability, fitness costs, and modes of resistance, including genetics, biochemical, and molecular mechanisms.

We reviewed 63 studies of laboratory resistance published in peer-reviewed journals for 11 species of major lepidopteran pests on cotton and corn that were targeted by 15 *Bt* toxins during the years 2000–2018 (Table 2). We searched the literature by using PubMed and Google Scholar, checking the bibliographies of all articles found with the keywords of laboratory-selection, *Bt* resistance, *Bt* toxins, and lepidopteran pests.

*Plutella xylostella* developed very high resistance to *Bt* kurstaki, Cry1Ac, Cry1Ab, and Cry1Ca after 5–7 generations of selection in Malaysia [61,62,63,64,65,66], and to Cry1C and Cry1Ac after 7–26 generations in China [28,67,68,69]. Very high resistance was reported in China for *P. gossypiella* to Cry1Ac [5] and to Cry1Ab, Cry1F, and Cry1Ah after 35–49 generations [70,71,72]. In the same country, moderate resistance to Cry1Ab and high resistance to Cry1Ac occurred in *O. furnacalis* [73]; very high resistance to Cry1Ac after 28 [74,75,76,77], 87 [78], 10 [79], 125 [80], and 6 generations [81]; and to Cry2Ab after 37 generations in *H. armigera* [82]. In contrast, after 29 generations, *H. armigera* presented very low resistance to Cry2Ab [80].

In the USA’s lepidopteran populations, very high resistance to Cry1Ac was observed after 16 [83] and 3–65 generations [84,85,86,87] for *P. xylostella*; and Cry2Ab after 17 generations [88] for *P. gossypiella*. Additionally, the following resistances have been reported: very high resistance to Cry1Ab after 95 [89], and 56 generations [90], and to Cry1F after 53 generations [91], and very low resistance after 10 generations [92] in *O. nubilalis*; very high resistance to Cry1Ac after 9–11 generations [93,94], moderate–high resistance to MPVII [42] in *H. zea*, very high resistance to Cry1Ac and Cry2Aa after 24 generations [95] and to Vip3Aa after 13 generations [96] in *H. virescence*; very high resistance to Cry1Ab, Cry1F and Vip3A [48,97,98,99,100], moderate resistance to Cry1Ab, Cry1F and Cry2Ab2 [101,102,103], and low resistance to Cry2Ab2 [46,104] in *D. saccharalis* and *S. frugiperda*.

High resistance to Cry1Ac after eight generations [105], very high resistance to Cry1Ac after 15 generations [106] in *P. gossypiella*; low resistance to Cry1Ac after five generations [107], high resistance after 10–19 generations [108,109], and very high resistance after 15 generations [110] in *H. armigera* have been reported for Indian populations. 

In Australia, *H. armigera* and *H. punctigera* presented very high resistance to Cry1Ac after 21 generations [111,112]. In *H. armigera*, very high resistance to Cry1Ac was detected after five generations from Pakistan [24], and to Cry2Ab after 16 generations in Australia [113]. Low resistance to Cry1Ab was reported after 12 generations in *Mythimna unipuncta* in Spain [114]; and very high resistance to Cry1A.105 after 10 generations [115], Vip3Aa20 after 5 generations [116], and Cry1F after 5–11 generations [117,118] was reported in *S. frugiperda* in Brazil.

These studies indicate that the proportion of individuals carrying the resistance alleles increases according to *Bt* toxin exposure. These studies also describe that laboratory-selected strains exhibited higher level of resistance than those from the field, which may be due to population strains under continuous selection pressure. So, selection pressure may increase the rate and frequency of resistance development [119]. All non-behavioral resistance mechanisms involve changes in physiology that are the result of selection for resistance alleles [120].

**Table 2:** Laboratory-selected resistance to *Bacillus thuringiensis* toxins in different lepidopteran pests from 2000–2018.

Species	Country	Toxin	G *	RR ^a^	RL ^b^	Reference
*Plutella xylostella*	Malaysia	*Bt* kurstaki	5	112	Very high	[66]
	Malaysia	Cry1Ac	5	>10,500	Very high	[65]
		Cry1Ab	5	500	Very high	
	China	Cry1C	26	63,100	Very high	[67]
	Malaysia	Cry1Ac	7	209,000	Very high	[61]
		Cry1Ab	7	7700	Very high	
		Cry1Ca	7	273	Very high	
	China	Cry1Ac	8	47,500	Very high	[28]
	USA	Cry1Ac	16	>40,000	Very high	[83]
	Malaysia	Cry1Ac	NA	>5710	Very high	[62]
	Malaysia	Cry1Ab	NA	2455	Very high	[63]
	Malaysia	Cry1Ac	6	159	Very high	[64]
	China	Cry1Ac	20	1200	Very high	[68]
	China	Cry1Ac	7	4850	Very high	[69]
*Pectinophora gossypiella*	USA	Cry1Ac	3	>100	Very high	[84]
	USA	Cry1Ac	31	3100	Very high	[85]
	USA	Cry1Ac	65	1700	Very high	[86]
	USA	Cry2Ab	17	240	Very high	[88]
	USA	Cry1Ac	11	240	Very high	[87]
	India	Cry1Ac	8	64	High	[105]
	India	Cry1Ac	15	257	Very high	[106]
	China	Cry1Ac	NA	220	Very high	[5]
*Ostrinia nubilalis*	USA	Cry1Ab	10	10	Very low	[92]
	USA	Cry1Ab	95	2000	Very high	[89]
	USA	Cry1F	53	>3000	Very high	[91]
	USA	Cry1Ab	56	4278	Very high	[90]
*Ostrinia furnacalis*	China	Cry1Ab	35	107	Very high	[70]
	China	Cry1Ab	NA	39	Moderate	[73]
		Cry1Ac	NA	79	High	
	China	Cry1F	49	>1754	Very high	[71]
	China	Cry1Ah	48	200	Very high	[72]
*Helicoverpa zea*	USA	Cry1Ac	11	123	Very high	[93]
	USA	Cry1Ac	9	560	Very high	[94]
	USA	MVPII	9	33.9	Moderate	[42]
		Cry1Ac	9	57	High	
*Heliothis virescens*	USA	Cry1Ac	24	289	Very high	[95]
		Cry2Aa	24	>250	Very high	
	USA	Vip3Aa	13	2040	Very high	[96]
*Helicoverpa armigera*	Australia	Cry1Ac	21	321	Very high	[112]
	China	Cry1Ac	28	564	Very high	[74]
	Australia	Cry2Ab	16	6830	Very high	[113]
	India	Cry1Ac	5	13	Low	[107]
	China	Cry1Ac	87	2893	Very high	[78]
	China	Cry1Ac	28	438	Very high	[75]
	India	Cry1Ac	10	72	High	[108]
	India	Cry1Ac	15	257	Very high	[110]
	India	Cry1Ac	19	72	High	[109]
	China	Cry1Ac	28	540	Very high	[76,77]
	Pakistan	Cry1Ac	5	5440	Very high	[37]
	China	Cry1Ac	10	1200	Very high	[79]
	China	Cry1Ac	125	1000	Very high	[80]
		Cry2Ab	29	5.6	Very low	
	China	Cry2Ab	37	130	Very high	[82]
	China	Cry1Ac	6	220	Very high	[81]
*Helicoverpa punctigera*	Australia	Cry1Ac	21	113	Very high	[111]
*Mythimna unipuncta*	Spain	Cry1Ab	12	11	Low	[114]
*Diatraea saccharalis*	USA	Cry1Ab	7	102	Very high	[100]
	USA	Cry1Ab	NA	43.4	Moderate	[103]
	USA	Cry1Ab	NA	>100	Very high	[99]
	USA	Cry1Ab	NA	>526	Very high	[48]
	USA	Cry2Ab2	NA	>11	Low	[104]
*Spodoptera frugiperda*	USA	Cry1F	NA	>34.6	Moderate	[102]
	USA	Cry1F	NA	>289	Very high	[98]
	Brazil	Cry1A.105	10	>3368	Very high	[115]
	Brazil	Vip3Aa20	5	>3200	Very high	[116]
	Brazil	Cry1F	11	>183	Very high	[117]
	Brazil	Cry1F	5	307	Very high	[118]
	USA	Cry2Ab2	NA	>15	Low	[46]
	USA	Cry2Ab2	NA	>26	Moderate	[101]
	USA	Vip3A	NA	>632	Very high	[97]

* Number of generations selected for resistance. ^a^ Resistance ratio, calculated as LC_50_ of tested strain/LC_50_ of susceptible strain. ^b^ Resistance level.

## 5. Cross-Resistance to Other *Bt* Toxins

Cross-resistance is defined as resistance due to a single mechanism and/or mode of action that provides resistance to a different insecticide. We found 37 cases of cross-resistance for nine species of major lepidopteran pests of cotton and corn that were targeted by 28 *Bt* toxins from 2000 to 2018.

### 5.1. Very High and High Cross-Resistance to Other Bt Toxins

In the USA, very high cross-resistance to Cry1Aa, Cry1Ab, Cry1Ac, and Cry1F in the Cry1Ab-R and Cry2Ab-R strains of *P. gossypiella* [88,121], and to Cry1Aa, Cry1Ab, Cry1F, and Cry1J in the Cry1Ac-R strain of *P. xylostella* [28] was observed. Similarly, there were very high levels of resistance to Cry1Aa and Cry1Ac in the Cry1Ab-R strains of *O. nubilalis* and *D. saccharalis* [48,90]. For *H. virescens*, a strain that was resistant to Cry1Ac subsequently selected for resistance to Cry2Aa showed increased resistance to Cry1Ac [95] (Table 3). 

In China, Cry1Ab and Cry1Ah resistant strains of *O. furnacalis* showed very high cross-resistance to Cry1Ah, CryA1c [70,73], and Cry1F [72]. Cry1Ac-resistant strains of *H. armigera* revealed very high cross-resistance to Cry1Aa [74,79]. The ACry1Ac-R strain of *P. xylostella* presented very high cross-resistance to Cry1Ab, Cry1F, Cry1J, Cry1C, and Cry1Ac+Cry1C in Malaysia [64], while a *H. armigera* strain resistant to Cry1Ac and Cry2Ab exhibited cross-resistance to Cry1Ab and Cry2Aa in Australia [112,113], and a Cry1F-R strain of *S. frugiperda* to Cry1A.105 in Brazil [115] (Table 3).

In Malaysia, high cross-resistance to Cry1Ac was observed in the Cry1Ab-R, *Bt* kurstaki-R, and *Bt* Aizawai-R strains of *P. xylostella* [65]. The Cry1Ab-R strains of *O. nubilalis* and *D. saccharalis* exhibited high cross-resistance to Cry1Ac and Cry1Aa, respectively, and a Cry1Ac-R strain of *H. zea* showed high cross-resistance to Cry1A.105 in the USA [42,92,99]. In China, high cross-resistance to Cry1Ab, Cry1Ac, and Cry2Aa in the Cry1Ac-R and Cry2Ab-R strains of *H. armigera* were reported [79,80,82] (Table 3).

All together, these findings reveal that different pest strains resistant to a *Bt* toxin may develop cross-resistance to different toxins, indicating possible multiple resistances.

### 5.2. Moderate and Low Level of Cross-Resistance to Other Bt Toxins

The moderate cross-resistance to Cry1Ac was reported in a Cry1Ab-R strain of *P. xylostella* from Malaysia, to Cry1Ac in a Cry1Ab-R strain of *D. saccharalis* [99], to Cry1Ab, Cry1Ac in a Cry1F-R strain of *S. frugiperda* [102], and Cry1Ab in a Cry1Ac-R strain of *H. zea* [42] from the USA. Moderate cross-resistance was conferred in Cry1Ab-R, Cry1F-R, and Cry1Ah-R strains of *O. furnacalis* to Cry1Ac [70], Cry1F, Cry1Ab, and Cry1Ac [70,71,72,73], and a Cry1Ac-R strain of *H. armigera* to Cry1Ab, and Cry1Aa [74,75,82] in China (Table 4).

A low level of cross-resistance was observed in China for Cry1Aa, Cry1Ab and Cry1F in a Cry1Ac-R strain of *P. xylostella* [68], to Cry1F in a Cry1Ac-R strain of *O. furnacalis* [73], to Cry1Aa, and Cry1Ab in a Cry2Ab-R strain of *H. armigera* [82]. A *Bt* kurstaki-R strain of *P. xylostella* from Malaysia showed a low level of cross-resistance toCry1Ab [65]. Similarly, Cry1F-R and Cry2Ab2-R strains of *S. frugiperda* also exhibited low level of cross-resistance to Cry2Ab2 and Cry2Ae in Brazil and the USA, respectively [101,115] (Table 4).

### 5.3. Very Low Level of Cross-Resistance to Other Bt Toxins

The very low level of cross-resistance to Cry1Ab, *Bt* kurstaki, *Bt* Aizawai, Cry1Ca, Cry1Da, Cry1Ac, Cry9Aa, Cry9C, and Cry1Bb were reported for Cry1Ac-R, Cry1Ab-R, *Bt* Aizawai-R, Cry1Ca-R, and Cry1Da-R strains of *P. xylostella* in Malaysia [61,64,65]. Similar findings were reported for a Cry1C-R strain of *P. xylostella* for cross-resistance to Cry1Bb, Cry9Aa, and Cry9C in the USA [28]. Also in the USA, a Cry1Ab-R strain of *D. saccharalis* showed very low cross-resistance to Cry1A.105, and Cry1F [48,99], while a Cry1Ab-R strain of *O. nubilalis*, a Cry1Ac-R strain of *H. zea*, and a Vip3A-R strain of *S. frugiperda* showed very low levels of cross-resistance to Cry1F [90,92], MVPII, Cry2Ab, and Cry1Fa [42,93,94], and to Cry1F and Cry2Ab2 [97], respectively. Very low cross-resistance to *Bt* kurstaki and Cry1C was reported in a Cry1Ac-R strain of *P. xylostella* [68], to Cry2Ab in a Cry1Ac-R strain of *P. gossypiella* [5], and to Cry1F, Cry1Ac, and Cry1Ah in Cry1Ab-R, Cry1Ac-R, and Cry1F-R strains of *O. furnacalis* in China [70,71,73]. Very low cross-resistance in a Cry1Ac-R strain of *H. armigera* was found in *Bt* kurstaki and Cry2Ab in China [74,79,80], and Cry1Aa and Cry1Ab in India [107] (Table 5).

### 5.4. No Cross-Resistance to Other Bt Toxins

No cross-resistance was observed to Cry1Aa, Cry1Bb, Cry1Ca, Cry1Da, Cry1Ea, and Cry1Ja in a Cry1Ac-R strain of *P. gossypiella* [122], to Cry1D in a Cry1C-R strain of *P. xylostella* [16], and to Cry2Ab2 and Cry9C in Cry1Ab-R strains of *D. saccharalis* and *O. nubilalis* in the USA [83,90]. Similarly, no cross-resistance was seen to Vip3Aa and Cry2Aa2 in Cry1Ac-R strains of *H. zea* [30,84], to Vip3Aa in a Cry1F-R strain of *S. frugiperda* [89], to Cry1F, Cry1A.105, and Vip3A in Cry2Ab2-R [92] *S. frugiperda*, and Cry2Ae in a Vip3A-R *S. frugiperda* [88] strain. In China, no cross-resistance to Cry1B or Cry2Aa was observed in a Cry1Ac-R strain of *P. xylostella* [59], to Cry1Ie in Cry1Ab-R, Cry1Ac-R, and Cry1Ah-R strains of *O. furnacalis* [61,63,64], and to Cry2Aa or Vip3A11 in Cry1Ac-R, and Cry1Ab-R strains of *H. armigera* [20,65,66]. Similarly, a lack of cross-resistance was reported in a Cry1Ac-R strain of *P. gossypiella* to Cry2Ab2 from India [123], the Cry1Ac-R and Kurstaki-R strains of *P. xylostella* to *Bt* Aizawai and Cry1D from Malaysia [55,56], and in Cry1Ac-R, Cry2Ab-R, and Vip3Aa-R strains of *H. armigera* to Cry2Aa, Cry2Ab, Cry1Ac, and Dipel^®^ from Australia [103,104,124] (Table 6).

## 6. Mechanisms of *Bt* Resistance

Variation in any step of the toxin mode of action may result in reduced susceptibility to *Bt* toxins. This also contributes to conferring cross-resistance among toxins [125]. Therefore, the success of resistance management is also dependent upon the biochemical mechanisms of resistance to detect the resistance genes in the field populations. There are different mechanisms involved in the evolution of *Bt* resistance, including reduced binding sites, mutated binding sites, altered proteolysis, or even behavioral changes. These multiple mechanisms of resistance correspond to genetic changes for insect survival after insecticide exposure. Thus, resistance to the insecticide can be expressed in the next generation [126]. Fifty-seven studies of 13 *Bt resistant* lepidopteran species have demonstrated different mechanisms of resistance to 11 *Bt* toxins (Table 7).

In the USA, reduced binding site mechanisms of resistance to Cry1C, Cry1Ac, Cry1Ab, Cry1Fa, and Cry2Ae were observed for *H. virescens*, *H. zea*, *H. armigera*, *P. gossypiella,* and *P. xylostella* [67,122,123,127]. The resistance to Cry1Ac, Cry1Ab, Cry1Aa, and Cry1Fa for *H. armigera* and *H. virescens* [90,95,124], *O. nubilalis* [90], *P. gossypiella* [85], and *S. frugiperda* [124,128] was due to reduced binding site mechanisms. The mechanism of resistance to Cry1Ac was attributed to mutations in an ABC membrane transporter protein for *H. virescens* [129]. Cadherin-like mutations were linked to resistance to Cry1A, Cry1Ab, Cry1Ac, and Cry1F for *Manduca sexta*, *O. nubilalis*, *P. gossypiella*, and *T. ni* [130,131,132,133,134,135,136,137]. Altered receptor binding (aminopeptidases-N) was linked to resistance to Cry1Ac and Cry1Ab for *O. nubilalis* and *T. ni* [138,139], a *SfABCC2* gene mutation was linked to resistance to Cry1Fa for *S. frugiperda* [140], unaltered binding was observed for resistance to Cry1Ca for *P. xylostella* [141] and multiple mechanisms were reported for resistance to Cry1Ab for *O. nubilalis* [83], and Cry2Ab and Cry1Ac resistance for *P. gossypiella* [79,122].

In China, cadherin gene and aminopeptidase-N mutations were linked to resistance to Cry1Ac [74,81,142,143], multiple mechanisms were linked to resistance to Cry1Ac [76,77,144], and reduced binding sites were linked to resistance to Cry1Ac [145] for *H. armigera*. Cadherin-like mutations were linked to resistance to Cry1Ac, and Cry2Aa for *P. gossypiella* [5], *S. exigua* [146,147], altered expression of *ALP* and *ABCC* genes were linked to resistance [148] to Cry1Ac for *P. xylostella*.

Alteration in the toxin binding sites was linked to resistance to Cry1Ja, Cry1Ac, and Cry1A.105, Cry1Aa, Cry1Ab, and Cry1Fa for *H. armigera*, *M. sexta*, *O. nubilalis*, and *P. gossypiella* [149,150,151]; ABCC2 mutation was linked to resistance to Cry1Fa for *P. xylostella* [152]; altered, unaltered, and reduced bindings sites were reported for resistance to Cry1Ab, Cry1Ac, and Cry1Ab, respectively, for *P. interpunctella* [153] in Spain. Amino acid mutation to Cry1Ab [154], ABCC2 protein to Cry1A and Cry1F [155] for *Bombyx mori* in Japan; unspecific/altered bindings to Cry1Ac [112,156], unaltered specific binding to Vip3Aa [157] for *H. armigera*, cadherin-like mutation to Cry1Ac for *P. interpunctella* [111] in Australia; multiple mechanism to Cry1Ac for *H. armigera* in Pakistan [37]; cadherin-like gene disruption or reduced binding to Cry1Ac for *P. gossypiella* in India [105,158] were reported. In Malaysia, reduced/shared binding, multiple resistance mechanisms to Cry1Ac, *Bt* kurstaki, and Cry1Ab for *P. xylostella* [61,64,65,66]; cadherin-like gene to Cry1C for *S. exigua* in Korea [159] and reduced binding to Cry1Fa for *S. frugiperda* in Brazil [53] were reported as resistance mechanisms.

These findings reveal that different insects have different mechanisms, including multiple mechanisms of *Bt* toxins, indicating that further studies are necessary for the comprehension of the molecular resistance mechanisms in different species and populations.

**Table 7:** Mechanisms conferring resistance to *Bacillus thuringiensis* toxins in different lepidopteran pests from 2000–2018.

Species	Country	Toxin	Resistance Mechanism	Reference
*Bombyx mori*	Japan	Cry1Ab	Amino acid mutation	[154]
	Japan	Cry1A, Cry1F	ABCC2 protein	[155]
*Helicoverpa armigera*	Australia	Cry1Ac	Reduced binding sites	[112]
	China	Cry1Ac	Disruption of cadherin gene	[74]
	China	Cry1Ac	Mutation in the cadherin alleles	[142]
	China	Cry1Ac	Aminopeptidase-N mutation	[143]
	USA	Cry1Ac	Reduced binding sites	[124]
	Pakistan	Cry1Ac	Multiple mechanism	[37]
	China	Cry1Ac	Multiple mechanisms	[76,77,144]
	China	Cry1Ac	Reduced binding sites	[145]
	Australia	Vip3Aa	Unaltered binding sites	[157]
	China	Cry1Ac	Cadherin mutation	[81]
*Heliothis virescens*	USA	Cry1Aa	Reduced binding sites	[95]
	USA	Cry1Ac	ABC protein mutations	[129]
	USA	Cry1Ac	Reduced binding sites	[124]
	USA	Cry2Ae	Reduced binding sites	[122]
*Manduca sexta*	USA	Cry1A	Cadherin mutation	[136]
	USA	Cry1Ab	Low cadherin expression	[137]
*Ostrinia nubilalis*	USA	Cry1Ab, Cry1Ac	Altered binding sites	[160]
	USA	Cry1Ab	Multiple resistance mechanisms	[92]
	USA	Cry1Ab	Altered receptor binding	[138]
	USA	Cry1Ab, Cry1Aa	Reduced binding sites	[90]
*Pectinophora gossypiella*	USA	Cry2Ab	Multiple mechanisms	[88]
	USA	Cry1Ac	Multiple mechanisms	[121]
	USA	Cry1Ac	Reduced binding sites	[85]
	USA	Cry1Ac	Mutation in cadherin gene	[132,133,134]
	India	Cry1Ac	Disrupted cadherin alleles	[158]
	India	Cry1Ac	Reduced binding sites	[105]
	China	Cry1Ac	Cadherin mutation	[5]
*Plodia interpunctella*	Spain	Cry1Ab	Slightly altered binding sites	[153]
		Cry1Ac	Unaltered binding sites	
		Cry1Ab	Reduced binding sites	
		Cry1A	Altered binding sites	
	Australia	Cry1Ac	Cadherin mutation	[111]
*Plutella xylostella*	USA	Cry1Ca	Unaltered binding sites	[141]
	USA	Cry1C	Reduced binding sites	[67]
	Malaysia	Cry1Ac	Reduced binding sites	[66]
	Malaysia	Cry1Ab	Reduced binding sites	[65]
	Malaysia	Cry1Ac	Multiple resistance mechanisms	[64]
	Malaysia	Cry1Ac	Altered binding sites	[61]
	Malaysia	*Bt* kurstaki	Multiple resistance mechanisms	[66]
	China	Cry1Ac	Reduced binding sites	[68]
	Spain	Cry1Fa	ABCC2 mutation	[161]
	China	Cry1Ac	Multiple mechanisms	[152]
	China	Cry1Ac	Altered expression of ALP and ABCC genes	[148]
*Spodoptera exigua*	Korea	Cry1C	Cadherin gene mutation	[159]
	China	Cry1Ac	Cadherin gene mutation	[146]
	China	Cry1Ac/Cry2Aa	Cadherin gene mutation	[147]
*Spodoptera frugiperda*	USA	Cry1Fa	Reduced binding sites	[124]
	Brazil	Cry1Fa	Reduced binding sites	[53]
	USA	Cry1Fa	Reduced binding sites	[128]
	USA	Cry1Fa	Mutation in the *SfABCC2* gene	[140]
*Trichloplusia ni*	USA	Cry1Ac	Altered binding sites	[162]
	USA	Cry1Ac	Alterationof aminopeptidases-N	[139]
	USA	Cry1Ac	Cadherin gene, aminopeptidase-N, *ABCC2* gene	[130,131]

## 7. Inheritance of Resistance to *Bt* Toxins

The expression of resistant genes in heterozygotic individuals can confer dominance of resistance to insecticides [23]. If resistance is completely dominant, only one parental individual needs to possess the trait for it to be fully expressed in the offspring. Thus, completely dominant resistance alleles rapidly become established in the populations, which are hard to manage. If resistance is incompletely dominant, the trait will be partially expressed in heterozygous offspring. This type of resistance can be managed with higher expression of the toxin or rotational uses of different *Bt* toxins where there is no cross-resistance [163]. If the resistance is completely recessive, only offspring that are homozygous for the resistance allele will be resistant and the resistance cannot rapidly be established in the population if appropriate management practices are followed, because the persistence of heterozygotes may be promoted (e.g., by alternative hosts or refuge) and they may be easily killed under field conditions.

We reviewed 58 cases of resistance inheritance that were published in peer-reviewed journals for 11 species of major lepidopteran pests of cotton and corn that have been targeted by 15 *Bt* toxins from 2000 to 2018 (Table 8). We searched the literature by using PubMed and Google Scholar, checking the bibliographies of all articles found with the keywords of genetics, *Bt* resistance, inheritance, and dominance.

Among the 58 cases, only one case of complete dominance of resistance in *O. furnacalis* was reported in China and all other reports showed completely recessive to incompletely dominant resistance to different *Bt* toxins. In all cases of resistance to *Bt* toxins studied, the inheritance of resistance was autosomal. However, there were a few examples where the sex of the resistant parents had a significant influence, such as in the Cry1Ac-SEL and Cry1Ab-SEL strains of *P. xylostella* [61,63,164], *H. armigera*, and *P. gossypiella* [106,110]. Incompletely recessive and monogenic resistance to *Bt* kurstaki [66], completely recessive/incompletely dominant and monogenic/polygenic resistance to Cry1Ac [61,62,65,164], and incompletely dominant and polygenic resistance to Cry1Ab [63] for *P. xylostella* have been reported in Malaysia. 

Lepidopteran pest populations in China were found to possess incompletely recessive and polygenic resistance to Cry1C [67], incompletely recessive and monogenic resistance to Cry1Ac for *P. xylostella* [68,69], and incompletely/completely recessive and monogenic/polygenic resistance to Cry1Ac for *H. armigera* [74,75,78]. Incompletely dominant resistance to Cry1Ac [77,79], incompletely dominant and polygenic resistance to Cry2Ab [82] for *H. armigera*, completely recessive resistance to Cry1Ac for *P. gossypiella* [5], completely dominant and polygenic resistance to Cry1Ab and Cry1Ac, incompletely dominant and polygenic resistance to Cry1F, and incompletely recessive and polygenic resistance to Cry1Ah for *O. furnacalis* have been reported [71,72,73]. 

In the USA, various types of resistance have been reported, including completely recessive and monogenic resistance to Cry1Ab and Cry1F for *O. nubilalis* [89,91], incompletely dominant resistance to Cry1Ac [84], incompletely recessive/completely recessive and monogenic resistance to Cry1Ac [85,158], and completely recessive resistance to Cry2Ab [88] for *P. gossypiella,* incompletely recessive and monogenic resistance to Cry1Ac for *Trichoplusia ni* [165], incompletely recessive, and monogenic resistance to Cry1Ab [103,166], and incompletely dominant and monogenic resistance to Cry2Ab2 [104] for *D. saccharalis*. In addition, incompletely recessive/completely recessive, and monogenic resistance to Cry1F, Cry2Ab2, Cry1A, and Vip3A have been reported in *S. frugiperda* in the USA, Brazil, and Argentina [97,98,102,167,168,169].

Incompletely/completely recessive, incompletely dominant, and monogenic resistance to Cry1Ac for *H. armigera* [107,109,110,170] and incompletely recessive resistance to Cry1Ac for *P. gossypiella* was reported in India [106]. In Brazil, there was incompletely recessive and monogenic resistance to Cry1A and Cry1F [115,117,118,171] as well as completely recessive and monogenic resistance to Cry1A+Cry2Ab, Vip3Aa20, and Cry1A+Cry1F+Cry2Ab2 [116,172,173]. 

Incompletely/completely recessive and monogenic resistance to Cry1Ac, Cry2Ab, and Vip3Aa for *H. armigera and H. punctigera* were reported in Australia [111,112,113,157,174]. Incompletely dominant and monogenic resistance to Cry1Ac for *H. armigera* was reported in Pakistan [37], incompletely dominant and polygenic resistance to Cry1Ab for *M. unipuncta* was reported in Spain [114], incompletely dominant resistance to Cry1Ab for *B. fusca* was reported in South Africa [175], incompletely recessive and polygenic resistance to *Bt* kurstaki for *T. ni* was reported in Canada [176], and incompletely recessive resistance to Cry1F for *S. frugiperda* was reported in Argentina [177].

**Table 8:** Inheritance and type of resistance to *Bacillus thuringiensis* in different lepidopteran pests from 2000–2018.

Species	Country	Strain	Inheritance Type	Reference
*Plutella xylostella*	Malaysia	*Bt* kurstaki-R	Autosomal, incompletely recessive, monogenic	[66]
	Malaysia	Cry1Ac-R	Autosomal, incompletely dominant, monogenic	[65]
	China	Cry1C-R	Autosomal, incompletely recessive, polygenic	[67]
	Malaysia	Cry1Ac-R	Sex-linked, incompletely dominant, polygenic	[61]
	Malaysia	Cry1Ac-R	Sex-linked, incompletely dominant, polygenic	[164]
	Malaysia	Cry1Ac-R	Autosomal, completely recessive, monogenic	[62]
	Malaysia	Cry1Ab-R	Sex-linked, incompletely dominant, polygenic	[63]
	China	Cry1Ac-R	Autosomal, incompletely recessive, monogenic	[68]
	China	Cry1Ac-R	Autosomal, incompletely recessive, monogenic	[69]
*Helicoverpa armigera*	Australia	Cry1Ac-R	Incompletely recessive	[112]
	China	Cry1Ac-R	Autosomal, incompletely recessive, monogenic	[74]
	India	Cry1Ac-R	Autosomal, incompletely dominant, monogenic	[170]
	Australia	Cry2Ab-R	Autosomal, completely recessive, monogenic	[113]
	India	Cry1Ac-R	Autosomal, incompletely recessive	[107]
	China	Cry1Ac-R	Autosomal, incompletely recessive, polygenic	[78]
	China	Cry1Ac-R	Autosomal, completely recessive	[75]
	India	Cry1Ac-R	Sex-linked, incompletely dominant, monogenic	[110]
	India	Cry1Ac-R	Autosomal, completely recessive, monogenic	[109]
	China	Cry1Ac-R	Autosomal, incompletely dominant	[77]
	Pakistan	Cry1Ac-R	Autosomal, incompletely dominant, monogenic	[37]
	China	Cry1Ac-R	Autosomal, incompletely dominant	[79]
	Australia	Vip3Aa-R	Completely recessive, monogenic	[157]
	China	Cry2Ab-R	Autosomal, incompletely dominant, polygenic	[82]
	China	Cry1Ac-R	Autosomal, recessive	[81]
*Helicoverpa punctigera*	Australia	Cry2Ab-R	Autosomal, completely recessive, monogenic	[174]
	Australia	Cry1Ac-R	Completely recessive, monogenic	[111]
*Mythimna unipuncta*	Spain	Cry1Ab-R	Autosomal, incompletely dominant, polygenic	[114]
*Ostrinia nubilalis*	USA	Cry1Ab-R	Autosomal, monogenic	[89]
	USA	Cry1F-R	Autosomal, completely recessive, monogenic	[91]
*Pectinophora gossypiella*	USA	Cry1Ac-R	Autosomal, incompletely dominant	[178]
	USA	Cry1Ac-R	Autosomal, incompletely recessive, monogenic	[85]
	USA	Cry2Ab-R	Autosomal, completely recessive	[88]
	USA	Cry1Ac-R	Completely recessive, monogenic	[158]
	India	Cry1Ac-R	Autosomal, incompletely recessive	[106]
	China	Cry1Ac-R	Autosomal, completely recessive	[5]
*Busseola fusca*	South Africa	Cry1Ab-R	Incompletely dominant	[175]
*Ostrinia furnacalis*	China	Cry1Ab-R, Cry1Ac-R	Completely dominant, polygenic	[73]
	China	Cry1F-R	Autosomal, incompletely dominant, polygenic	[71]
	China	Cry1Ah-R	Autosomal, incompletely recessive, polygenic	[72]
*Trichoplusia ni*	Canada	*Bt* kurstaki-R	Autosomal, incompletely recessive, polygenic	[176]
	USA	Cry1Ac-R	Autosomal, incompletely recessive, monogenic	[165]
*Diatraea saccharalis*	USA	Cry1Ab-R	Incompletely recessive, monogenic	[166]
	USA	Cry1Ab-R	Autosomal, incompletely recessive, monogenic	[103]
	USA	Cry2Ab2-R	Autosomal, incompletely dominant, monogenic	[104]
*Spodoptera frugiperda*	USA	Cry1F-R	Autosomal, incompletely recessive	[102]
	USA	Cry1F-R	Autosomal, completely recessive, monogenic	[98]
	Brazil	Cry1A	Autosomal, incompletely recessive	[115]
	Brazil	Cry1A+Cry2Ab-R	Autosomal, completely recessive	[172]
	Brazil	Cry1F-R	Incompletely recessive	[171]
	Brazil	Vip3Aa20-R	Autosomal, completely recessive, monogenic	[116]
	Brazil	Cry1F-R	Autosomal, incompletely recessive, monogenic	[117,118]
	Brazil	Cry1A+Cry1F+Cry2Ab2-R	Completely recessive	[173]
	USA	Cry2Ab2-R	Autosomal, incompletely recessive, monogenic	[167]
	USA	Cry1F-R	Autosomal, recessive, monogenic	[168]
	Argentina	Cry1F-R	Autosomal, incompletely recessive	[177]
	USA	Cry1A-R	Autosomal, incompletely recessive, monogenic	[169]
	USA	Vip3A-R	Autosomal, incompletely recessive, monogenic	[97]

## 8. Fitness Cost of Resistance to *Bt* Toxins

We reviewed 36 cases of fitness costs published in peer-reviewed journals for 12 species of lepidopteran pests of cotton and corn that were targeted by 11 *Bt* toxins from 2000 to 2018 (Table 9). In the USA, there were reports of no fitness cost of resistance to Cry1Ab and Cry2Ab2 for *D. saccharalis* [104,179,180], Cry1F for *O. nubilalis* [181], Cry1F,Cry1A.105, and Cry2Ab2 for *S. frugiperda* [167,169,182]; increased fitness cost of resistance to Cry1Ac for *H. zea* [183], and *P. gossypiella* [87,132,178,184], Cry1Ab and Cry1F for *O. nubilalis* [185,186] and *S. frugiperda* [187], *Bt* kurstaki for *P. interpunctella* [188], and Cry1C for *S. exigua* [189]. Increased fitness cost of resistance to Cry1Ac for *H. armigera* [78], *S. exigua* [146] and no fitness cost of resistance to Cry1Ac and Cry2Ab for *H. armigera* [75,82] and Cry1Ac for *P. xylostella* [69] were reported in China.

No fitness cost of resistance was found for Cry1Ab for *Busseola fusca* in South Africa [190], *M. unipuncta* in Spain [114], *P. xylostella* in Malaysia [191], and Cry1F for *S. frugiperda* in Brazil [192,193]. However, there was increased fitness cost of resistance to Cry1Ac and *Bt* kurstaki for *H. armigera* in Australia and Iran [194,195]; Vip3A for *H. virescence*, Cry1Ac for *P. xylostella* in United Kingdom [196,197,198] and Cry1F, Cry1Ab, Vip3Aa20, and Cry1A.105+Cry1F+Cry2Ab2 for *S. frugiperda* in Brazil [115,116,173,199].

**Table 9:** Fitness costs associated with *Bacillus thuringiensis* resistance in different lepidopteran pests from 2000–2018.

Species	Country	Toxin	Strain	Type of fitness cost	Reference
*Buseola fusca*	S. Africa	Cry1Ab	VAA10Bt-Bt	No fitness cost	[190]
*Diatraea saccharalis*	USA	Cry1Ab	Cry1Ab-R	No fitness cost	[179]
	USA	Cry1Ab	RR-43A_BC_, RR-L5B_BC_	No fitness cost	[180]
	USA	Cry2Ab2	Cry2Ab2-RR	No fitness cost	[104]
*Helicoverpa armigera*	Australia	Cry1Ac	Cry1A-R	Increased fitness cost	[194]
	China	Cry1Ac	BtRF87	Increased fitness cost	[78]
	China	Cry1Ac	SCD-r1	No fitness cost	[75]
	Iran	*Bt* kurstaki	*Bt* kurstaki	Increased fitness cost	[195]
	China	Cry2Ab	An2Ab	No fitness cost	[82]
*Heliothis virescens*	UK	Vip3A	Vip-Sel	Increased fitness cost	[196]
*Helivoverpa zea*	USA	Cry1Ac	Cry1Ac-R	Increased fitness cost	[183]
*Mythimna unipuncta*	Spain	Cry1Ab	MON810-R	No fitness cost	[114]
*Ostrinia nubilalis*	USA	Cry1Ab	KS, SKY	Increased fitness cost	[185]
	USA	Cry1F	Cry1F-R	Increased fitness cost	[186]
	USA	Cry1F	Cry1F-R	No fitness cost	[181]
*Pectinophora gossypiella*	USA	Cry1Ac	AZP-R, Hybrids	Increased fitness cost	[132,184]
	USA	Cry1Ac	APHIS-98R, AZP-R	Increased fitness cost	[178]
	USA	Cry1Ac	*Bt*4R	Increased fitness cost	[87]
*Plodia interpunctella*	USA	*Bt* kurstaki	*Bt*-R	Increased fitness cost	[188]
*Plutella xylostella*	Malaysia	Cry1Ac	Cry1Ac-R	No fitness cost	[191]
	UK	Cry1Ac	Cry1Ac-R	Increased fitness cost	[197]
	China	Cry1Ac	NIL-R	No fitness cost	[69]
	UK	Cry1Ac	Cry1Ac-R	Increased fitness cost	[198]
*Spodoptera exigua*	USA	Cry1C	Cry1C-R	Increased fitness cost	[189]
	China	Cry1Ac	Cry1Ac-R	Increased fitness cost	[146]
*Spodoptera frugiperda*	USA	Cry1Fa	Cry1Fa-R	No fitness cost	[182]
	Brazil	Cry1F	Cry1F-R	Increased fitness cost	[115]
	USA	Cry1F	RR-FL/PR	Increased fitness cost	[187]
	Brazil	Cry1F	R-Cry1F	No fitness cost	[192]
	Brazil	Cry1Ab	Cry1Ab-R	Increased fitness cost	[199]
	Brazil	Vip3Aa20	Vip-R	Increased fitness cost	[116]
	Brazil	Cry1A.105+Cry1F+Cry2Ab2	MON89034+TC150+NK603-R	Increased fitness cost	[173]
	USA	Cry2Ab2	Cry2Ab2-R	No fitness cost	[167]
	Brazil	Cry1Fa	Lab-RR	No fitness cost	[193]
	USA	Cry1A.105	Cry1A.105-R	No fitness cost	[169]

## 9. Conclusions

In 22 studies, a high cross-resistance, 17 studies, a moderate cross-resistance, 21 studies, a very low cross-resistance, and 22 studies, the absence of cross-resistance were observed. Most of the resistant populations showed recessive to incompletely dominant types of resistance. The absence of cross-resistance to different *Bt* toxins in different populations is compatible with the idea of multiple resistance mechanisms involved. In the case of the absence of cross-resistance and a recessive mode of inheritance, the pyramiding of two different toxin genes, refuges, or high doses helps delay the development of *Bt* resistance. The inheritance and cross-resistance patterns extremely affect the strategies for managing *Bt resistance*. A high-dose strategy only can slow resistance when the resistance is recessive; in another case, if the resistance is dominant, then this strategy accelerates the development of resistance.

Concisely, many *Bt* toxins show no/very low levels of resistance, a lack of cross-resistance, recessive inheritance, and increased fitness costs, hence they are still a good alternative to synthetic insecticides for the control of lepidopteran pests. Knowledge of different management practices in combination with the rate of resistance development is essential to develop successful resistance management strategies. In addition, evaluation of already implemented management practices and awareness of pest biology will surely lead to beneficial decisions for the management of *Bt* resistance. The midgut is an important site for insecticidal action; therefore, it is likely that future attempts to develop insecticidal compounds will increasingly use the insect midgut as a target organ. Consequently, the selective toxicity of different *Bt* toxins is determined by the receptors in the midgut. Most studies of the fitness costs of *Bt* resistance have been conducted on moths. More studies on economically important beetles are needed.

Although this review provides substantial data to understand the selection of strains resistant to *Bt* toxins, some factors with the potential to drive resistance remain poorly understood, including the different modes of application of the toxin in the field, how different climates can contribute to selection for resistance, whether different gut microbiota can contribute to resistance, and how differential gene expression might be evaluated as a potential marker of populations with the potential for developing resistance to the toxin.

### Summary Points

(1) Field- and laboratory-evolved resistance, cross-resistance, inheritance, mechanisms, and fitness costs of resistance to different *Bt* toxins are reported from 20 countries.
(2) The studies refer mainly to lepidopteran pests from the United States of America, followed by China, Brazil, India, Malaysia, Spain, and Australia.
(3) In 22 studies, the pest populations showed absence of cross-resistance to different *Bt* toxins.
(4) Recessive to dominant inheritances of *Bt* resistance were reported in lepidopteran pests from different countries.
(5) Multiple mechanisms of *Bt* resistance were reported in different selected strains of lepidopteran pests.
(6) No to increased fitness costs were observed in most of the lepidopteran pests.
(7) The results of field and laboratory resistance, cross-resistance, inheritance, mechanisms and fitness costs of resistance are advantageous for predicting the threat of future resistance and making effective strategies to sustain the effectiveness of *Bt* crops.

## Figures and Tables

**Table 3:** High to very high levels of cross-resistance to *Bacillus thuringiensis* toxins in resistant strains of different lepidopteran pests from 2000–2018.

Species	Country	Resistant Strain	Toxin	RR ^a^	Reference
Very high levels of cross-resistance to other *Bt* toxins in the different *Bt* resistant strains					
*Pectinophora gossypiella*	USA	Cry2Ab-R	Cry1Ac	420	[88]
*Plutella xylostella*	USA	Cry1C-R	Cry1Aa	395	[28]
			Cry1Ab	595	
			Cry1F	7890	
			Cry1J	13,100	
	Malaysia	Cry1Ac-R	Cry1Ab	595	[64]
			Cry1F	7890	
			Cry1J	13,100	
			Cry1C	1090	
			Cry1Ac+Cry1C	14,500	
*Ostrinia nubilalis*	USA	Cry1Ab-R	Cry1Aa	2775	[90]
			Cry1Ac	535	
*Diatraea saccharalis*	USA	Cry1Ab-R	Cry1Aa	193	[48]
			Cry1Ac	267	
*Ostrinia furnacalis*	China	Cry1Ab-R	Cry1Ah	131	[70]
	China	Cry1Ab-R	Cry1Ac	113	[73]
	China	Cry1Ah-R	Cry1F	464	[72]
*Helicoverpa armigera*	Australia	Cry1Ac-R	Cry1Ab	157	[112]
	China	Cry1Ac-R	Cry1Aa	103	[74]
	Australia	Cry2Ab-R	Cry2Aa	9640	[113]
	China	Cry1Ac-R	Cry1Aa	260	[79]
*Heliothis virescens*	USA	Cry1Ac/Cry2A-R	Cry1Ac	188	[95]
*Spodoptera frugiperda*	Brazil	Cry1F-R	Cry1A.105	207	[115]
High levels of cross-resistance to other *Bt* toxins in the different *Bt* resistant strains					
*Plutella xylostella*	Malaysia	Cry1Ab-R	Cry1Ac	60	[65]
		*Bt* kurstaki-R	Cry1Ac	100	
		*Bt* Aizawai-R	Cry1Ac	70	
*Ostrinia nubilalis*	USA	Cry1Ab-R	Cry1Ac	52.6	[92]
*Diatraea saccharalis*	USA	Cry1Ab-R	Cry1Aa	>80	[99]
*Helicoverpa armigera*	China	Cry1Ac-R	Cry1Ab	69	[79]
	China	Cry2Ab-R	Cry1Ac	61	[80]
	China	Cry2Ab-R	Cry2Aa	81	[82]
*Helicoverpa zea*	USA	Cry1Ac-R	Cry1A.105	51.3	[42]

^a^ Resistance ratio, calculated as LC_50_ of tested strain/LC_50_ of susceptible strain.

**Table 4:** Low to moderate levels of cross-resistance to *Bacillus thuringiensis* toxins in resistant strains of different lepidopteran pests from 2000–2018.

Species	Country	Resistant Strain	Toxin	RR ^a^	Reference
Moderate levels of cross-resistance to other *Bt* toxins in the different *Bt* resistant strains					
*Plutella xylostella*	Malaysia	Cry1Ab-R	Cry1Ac	40	[61]
*Diatraea saccharalis*	USA	Cry1Ab-R	Cry1Ac	45	[99]
*Spodoptera frugiperda*	USA	Cry1F-R	Cry1Ab	22	[102]
			Cry1Ac	35	
*Ostrinia furnacalis*	China	Cry1Ab-R	Cry1Ac	36	[70]
	China	Cry1Ab-R	Cry1F	49	[73]
	China	Cry1F-R	Cry1Ab	22.8	[71]
			Cry1Ac	26.9	
	China	Cry1Ah-R	Cry1Ab	28.38	[72]
			Cry1Ac	22.11	
*Helicoverpa armigera*	China	Cry1Ac-R	Cry1Ab	>46	[74]
	China	Cry1Ac-R	Cry1Ab	31	[75]
			Cry1Aa	41	
	China	Cry2Ab-R	Cry1Ac	22	[82]
*Helicoverpa zea*	USA	Cry1Ac-R	Cry1Ab	22.4	[42]
Low levels of cross-resistance to other *Bt* toxins in the different *Bt* resistant strains					
*Plutella xylostella*	Malaysia	*Bt* kurstaki-R	Cry1Ab	18	[65]
	China	Cry1Ac-R	Cry1Aa	13	[68]
			Cry1Ab	18	
			Cry1F	>16	
*Ostrinia furnacalis*	China	Cry1Ac-R	Cry1F	18	[73]
*Helicoverpa armigera*	China	Cry2Ab-R	Cry1Aa	20	[82]
			Cry1Ab	18	
*Spodoptera frugiperda*	Brazil	Cry1F-R	Cry2Ab2	11	[115]
	USA	Cry2Ab2-R	Cry2Ae	>10	[101]

^a^ Resistance ratio, calculated as LC_50_ of tested strain/LC_50_ of susceptible strain.

**Table 5:** Very low cross-resistance to *Bacillus thuringiensis* toxins in resistant strains of different lepidopteran pests from 2000–2018.

Species	Country	Resistant Strain	Toxin	RR ^a^	Reference
*Plutella xylostella*	Malaysia	Cry1Ac-R	Cry1Ab	5.0	[65]
			*Bt* kurstaki	4.0	
		Cry1Ab-R	*Bt* kurstaki	5.0	
			*Bt* Aizawai	2.0	
		*Bt* Aizawai-R	Cry1Ab	8.0	
			*Bt* Kurstaki	3.0	
		Cry1Ac-R	Cry1Ab	3.0	[61]
			Cry1Ca	2.0	
			Cry1Da	3.0	
		Cry1Ab-R	Cry1Ca	2.0	
			Cry1Da	2.0	
		Cry1Ca-R	Cry1Ac	4.0	
			Cry1Ab	3.0	
			Cry1Da	2.0	
		Cry1Da-R	Cry1Ac	2.0	
			Cry1Ab	2.0	
			Cry1Ca	2.0	
	USA	Cry1C-R	Cry1Bb	2.3	[28]
			Cry9Aa	3.0	
			Cry9C	3.5	
	Malaysia	Cry1Ac-R	Cry9Aa	3	[64]
			Cry9C	4	
			Cry1Bb	2	
	China	Cry1Ac-R	*Bt* kurstaki	2.8	[68]
			Cry1C	2	
*Diatraea saccharalis*	USA	Cry1Ab-R	Cry1A.105	4.1	[99]
			Cry1F	6.9	[48]
*Pectinophora gossypiella*	China	Cry1Ac-R	Cry2Ab	2.1	[5]
*Ostrinia nubilalis*	USA	Cry1Ab-R	Cry1F	3.4	[92]
	USA	Cry1Ab-R	Cry1F	5.8	[90]
*Ostrinia furnacalis*	China	Cry1Ab-R	Cry1F	6	[70]
	China	Cry1Ac-R	Cry1Ac	10	[73]
	China	Cry1F-R	Cry1Ah	3.7	[71]
*Helicoverpa armigera*	China	Cry1Ac-R	*Bt* kurstaki	5	[74]
	India	Cry1Ac-R	Cry1Aa	5.8	[107]
			Cry1Ab	5.0	
	China	Cry1Ac-R	Cry2Ab	5.9	[79]
	China	Cry1Ac-R	Cry2Ab	6.8	[80]
*Helicoverpa zea*	USA	Cry1Ac-R	MVPII	10	[93]
	USA	Cry1Ac-R	Cry2Ab	1.98	[94]
	USA	Cry1Ac-R	Cry1Fa	2.0	[42]
			Cry2Ab	3.3	
*Spodoptera frugiperda*	USA	Vip3A-R	Cry1F	3.5	[97]
			Cry2Ab2	5.0	

^a^ Resistance ratio, calculated as LC_50_ of tested strain/LC_50_ of susceptible strain.

**Table 6:** Absence of cross-resistance to *Bacillus thuringiensis* toxins in resistant strains of different lepidopteran pests from 2000–2018.

Species	Country	Resistant Strain	Toxin	RR ^a^	Reference
*Pectinophora gossypiella*	India	Cry1Ac-R	Cry2Ab2	0.38	[106]
*Plutella xylostella*	Malaysia	Cry1Ac-R	*Bt* Aizawai	1.0	[65]
		*Bt* Kurstaki-R	*Bt* Aizawai	1.0	
	USA	Cry1C-R	Cry1D	1.5	[28]
	Malaysia	Cry1Ac-R	Cry1D	1.5	[64]
	China	Cry1Ac-R	Cry1B	1.3	[68]
			Cry2Aa	1.2	
*Diatraea saccharalis*	USA	Cry1Ab-R	Cry2Ab2	0.5	[99]
*Ostrinia nubilalis*	USA	Cry1Ab-R	Cry9C	1.2	[92]
*Ostrinia furnacalis*	China	Cry1Ab-R	Cry1Ie	1.0	[70]
	China	Cry1Ab-R	Cry1Ie	1.6	[73]
		Cry1Ac-R	Cry1Ie	1.4	
	China	Cry1Ah-R	Cry1Ie	1.0	[72]
*Helicoverpa zea*	USA	Cry1Ac-R	Vip3A	0.9	[93]
			Cry2Aa2	1.6	
	USA	Cry1Ac-R	Vip3Aa	1.6	[42]
*Helicoverpa armigera*	Australia	Cry1Ac-R	Cry2Aa	1.0	[112]
			Cry2Ab	1.4	
	China	Cry1Ac-R	Cry2Aa	1.4	[74]
	Australia	Cry2Ab-R	Cry1Ac	1.5	[113]
			Dipel	0.8	
	China	Cry1Ac-R	Cry2Aa	1.2	[75]
	China	Cry1Ac-R	Vip3Aa11	1.0	[32]
		Cry1Ab-R		1.4	
*Spodoptera frugiperda*	USA	Cry1F-R	Vip3Aa	1.3	[98]
	USA	Cry2Ab2-R	Cry1F	1.4	[101]
			Cry1A.105	1.2	
			Vip3A	1.1	
	USA	Vip3A-R	Cry2Ae	1.1	[97]

^a^ Resistance ratio, calculated as LC_50_ of tested strain/LC_50_ of susceptible strain.

## Data Availability

No new data were created or analyzed in this study. Data sharing is not applicable to this article.
